# Attention-related impairment and contributing neuroinflammatory signalling in the prefrontal cortex of perinatal nicotine-exposed mice

**DOI:** 10.1017/neu.2025.2

**Published:** 2025-02-10

**Authors:** Sabide Duygu Uygun, Tansu Bilge Kose, Aslihan Bahadir-Varol, Burak Uzay, Emine Eren-Kocak

**Affiliations:** 1 Institute of Neurological Sciences and Psychiatry, Hacettepe University, Ankara, Turkey; 2 Department of Child and Adolescent Psychiatry, Faculty of Medicine, Ankara University, Ankara, Turkey; 3 Department of Psychiatry, Icahn School of Medicine at Mount Sinai, New York, USA; 4 Friedman Brain Institute, Nash Family Department of Neuroscience, Icahn School of Medicine at Mount Sinai, New York, USA; 5 Department of Psychiatry, Faculty of Medicine, Hacettepe University, Ankara, Turkey

**Keywords:** ADHD etiology, neuroinflammation, maternal smoking, perinatal nicotine exposure

## Abstract

**Objective:**

Previous studies on the aetiology of attention-deficit/hyperactivity disorder (ADHD) emphasise high heritability and the influence of maternal smoking during pregnancy, highlighting the role of gene–environment interactions. Additionally, low-grade peripheral inflammation is frequently observed in individuals with ADHD. However, the underlying neurobiological mechanisms remain unclear. We aimed to investigate neuroinflammatory signalling contributing to ADHD and explore behavioural and molecular changes in a mouse model.

**Methods:**

We examined neuroinflammatory signalling using a perinatal nicotine exposure (PNE) model via immunohistochemistry combined with cortical thickness (CT) measurement in the subregions of the prefrontal cortex (PFC). Mice were exposed to nicotine via drinking water containing 300 μg/ml nicotine and 2% sucrose starting 2 weeks before mating until weaning to induce ADHD-like symptoms, as opposed to controls receiving drinking water containing 2% sucrose alone. Behavioural tests were conducted to assess ADHD-like behaviours and accompanying anxiety on postnatal week 5. Inflammatory pathways in the anterior cingulate cortex (ACC), prelimbic cortex (PL), and infralimbic cortex (IL) were examined using Iba-1 and NF-κB immunolabelling, and microglial morphology was analyzed.

**Results:**

Findings showed increased CT, microglial cell number, activity, and NF-κB activation in the ACC, which correlated with attention-related impairment in PNE mice. Increased Iba-1 levels in the PL and IL, along with elevated NF-κB activation in the IL, were observed in PNE mice, which corresponded with a significant increase in anxiety-like behaviours compared to controls. PNE mice also morphologically exhibited microglia activation in all three subregions.

**Conclusion:**

PNE contributes to ADHD development through neuroinflammatory signalling, a common end pathway.

## Introduction

Attention deficit hyperactivity disorder (ADHD) is a prevalent neurodevelopmental condition with three clinical presentations: i) inattentive, ii) hyperactive-impulsive, and iii) combined. Meta-analyses show inattentive presentation is the most common across sexes (Ayano *et al*., 2023). There is a high comorbidity of anxiety disorders, with rates between 4 and 47% from preschool to adulthood (Krone and Newcorn, 2015). Factors influencing the causes, presentations, and treatment of ADHD symptoms can vary by sex (Carucci *et al*., 2023). While ADHD is more common in males, untreated females may also face significant challenges in school, family, and social settings. This underscores the need to consider sex differences in research and clinical practices to enhance interventions and outcomes for all individuals.

The neurobiological basis of ADHD is not fully understood (Cortese, 2012). Research shows that specific prefrontal cortex (PFC) subregions – including the dorsolateral, anterior cingulate, ventromedial, and orbitofrontal cortices – are linked to ADHD symptoms such as inattention, hyperactivity, and impulsivity, highlighting the importance of investigating subregion-specific changes (Cortese, 2012; Yu *et al*., 2023). Although findings regarding cortical thickness (CT) are conflicting (generally showing decreases), increased CT has also been reported in several cortical regions in ADHD patients, suggesting that delayed pruning and maturation may lead to attenuation of age-related decreases in CT (Levman *et al.*, 2022). ADHD has a high genetic heritability, though environmental factors also significantly influence its development, accounting for 10 to 40% of changes (Sciberras *et al*., 2017). Our understanding of these environmental influences is limited, particularly during the prenatal period. Epidemiological studies indicate that smoking during pregnancy increases ADHD risk in offspring (Faraone *et al*., 2021). Beyond this proposed causal relationship, maternal genetic liability for ADHD has also been associated with maternal smoking during pregnancy, raising further interest in the contribution of perinatal nicotine exposure (PNE) to ADHD development (Havdahl *et al*., 2022). PNE is associated with decreased dopamine turnover in the frontal cortex and striatum, volume reduction in the cingulate cortex, and ADHD-like behaviours in mice, suggesting a model for ADHD (Chan *et al*., 2020; Ugarte *et al*., 2023). Further research is essential to explore the molecular mechanisms linking PNE to ADHD development.

Perinatal risk factors may influence gene expression via epigenetic mechanisms that impact neuronal development and neurogenesis, often linked to maternal immune activation (Kim *et al*., 2020; Gustafsson *et al*., 2020; Cecil and Nigg, 2022). High concentrations of cytokines, chemokines, and oxidative stress markers were consistently demonstrated in peripheral blood and CSF samples of individuals with ADHD, as well as in tissue and serum samples of animal models of ADHD. Elevated levels of autoantibodies were reported in ADHD, along with a frequent cooccurrence of ADHD with various autoimmune and inflammatory disorders (Giana *et al*., 2015; Chuang *et al*., 2022). Besides, high gene polymorphisms associated with the inflammatory system in humans were associated with ADHD (Chan *et al*., 2016; Anand *et al*., 2017; Kozłowska *et al*., 2019). These findings suggest that low-grade peripheral inflammation plays a role in the pathophysiology of ADHD, albeit indirectly. Despite these findings, no studies to date have investigated inflammatory changes in the PFC subregions associated with ADHD symptoms in either human or animal brains.

During neurodevelopment, neuroinflammatory signalling, including but not limited to microglia, plays crucial roles in physiological processes essential for the development of neural circuits, such as programmed neuronal cell death, clearance of neural elements, support of neuronal survival, synapse elimination, and regulation of synapse formation (Cowan & Petri Jr, 2018). Therefore, disruptions in any element of neuroinflammatory signalling can interfer with normal synaptic pruning and brain development resulting in neurodevelopmental disorders (Mordelt & de Witte, 2023). There are several cellular and molecular components of the neuroinflammatory signalling. Among these, alarmins, which are released from microglia as well as other cells within the central nervous system in response to intrinsic threats, bind to pattern recognition receptors, leading to activation of the pro-inflammatory transcription factor NF-κB, which subsequently triggers the production of pro-inflammatory cytokines and chemokines (Bianchi, 2007; Zhang *et al*., 2023). This cascade increases microglial activity and number by upregulating the levels of alarmins and promoting nuclear NF-κB translocation in neurons, ultimately leading to suppression of neurogenesis (Uzay *et al*., 2024). Microglial activity plays a crucial role in the pruning and maturation of dendritic spines during development, as microglia ablation may lead to increased spine density (Pöpplau *et al*., 2024). Mice exposed to prenatal nicotine exhibit a lower density of dendritic spines in their CA1 pyramidal neurons compared to control mice. Notably, there is a significant increase in the proportion of thin (immature) spines and a decrease in the proportion of mushroom-type (mature) spines, indicating a developmental delay in the hippocampus of nicotine-exposed mice that resembles the characteristics seen in ADHD patients. In contrast, mice treated with a single dose of methylphenidate show no significant change in spine density among CA1 neurons compared to untreated mice. However, there is a substantial reduction in the proportion of immature spines and a significant increase in mature mushroom-type spines, suggesting that a single dose of methylphenidate promotes the maturation of dendritic spines in hippocampal neurons (Contreras *et al*., 2022). It remains to be elucidated whether inflammatory changes and microglial activity serve as mediators between PNE and its phenotypic consequences.

Our study aimed to investigate the role of neuroinflammation in the pathophysiology of ADHD using a PNE model in the juvenile period. We hypothesised that (i) during the juvenile period corresponding to postnatal week 5, the PNE group modelling ADHD would exhibit differences in prefrontal CT compared to controls, (ii) these differences would be accompanied by increased microglial activation driven by elevated NF-κB signalling, correlating with CT alterations, and (iii) subregion- and sex-specific differences in ADHD-like behaviours, CT, and neuroinflammatory signalling would be observed across the PFC. To test this, we examined neuroinflammatory signalling changes in distinct PFC subregions, including the anterior cingulate cortex (ACC), prelimbic cortex (PL), and infralimbic cortex (IL), using immunohistochemistry and CT measurements. We further investigated the correlation between these molecular changes and ADHD-like behavioural phenotypes, specifically hyperactivity, impulsivity, and attentional impairment, while also considering sex differences.

## Materials and methods

### PNE mouse model

**Figure 1.**
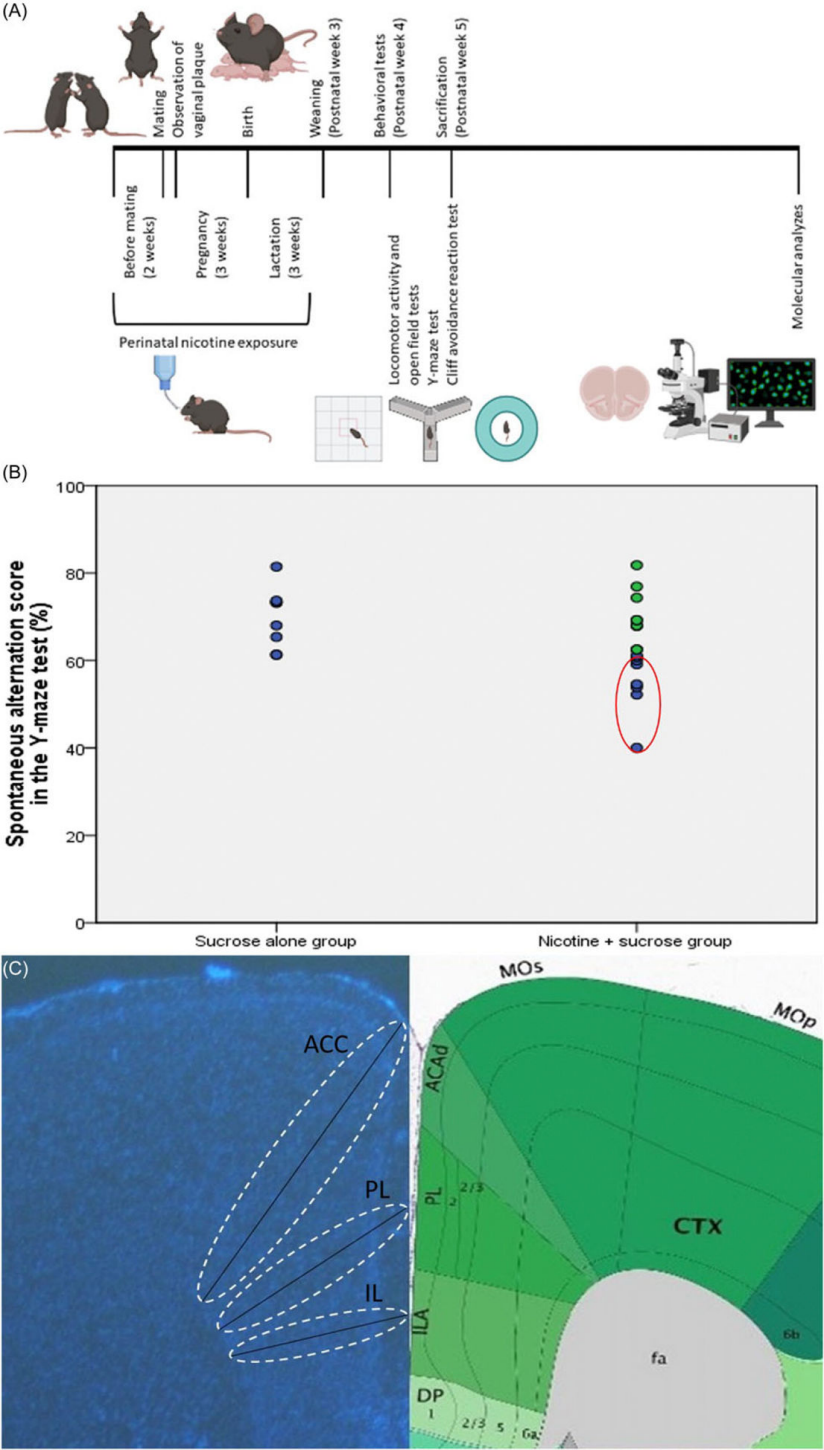
Perinatal nicotine exposure. (A) timeline illustrating perinatal nicotine exposure. (B) criteria used for selecting ADHD-modeling mice from those exposed. (C) measurement of cortical thickness in prefrontal cortex subregions (ACC: anterior cingulate cortex, PL: prelimbic cortex, IL: infralimbic cortex).

Twenty-four female and 12 male C57BL/6 mice (10–12 weeks old) were obtained from Hacettepe University Laboratory Animals Application and Research Center, Ankara. They were housed in a 12-hour light/dark cycle with ad libitum access to food and water. Female mice were randomly assigned to either the nicotine + sucrose group or the sucrose-alone group. Mice in the nicotine + sucrose group were provided with drinking water containing 300 μg/ml nicotine and 2% sucrose, while those in the sucrose-alone group were given drinking water containing only 2% sucrose (Polli *et al*., 2020). Sucrose was used as a sweetener to mask the bitter taste of nicotine in the drinking water. (Pauly *et al*., 2004; Bryden *et al*., 2016; Polli and Kohlmeier, 2018; Polli *et al*., 2020). The animals maintained healthy appearances and grooming. Following 2 weeks of these exposures, the female mice were mated with drug-naive male mice (two females were paired with one male per cage). When vaginal plugs were observed in female mice (indicating pregnancy), male mice were removed from the cages. Exposure of female mice in each experimental group to the specified drinking waters continued throughout pregnancy and lactation periods (Fig. 1A). Seventeen offspring were born from the female mice exposed to drinking water with nicotine and sucrose, and eight offspring born from those exposed to drinking water with only sucrose were maintained in their respective drinking waters until weaning. Male and female pups were weaned on the 21st postnatal day and housed in cages with 3–4 pups per cage. The drinking waters in cages of each experimental group were changed weekly. All control offspring generated during the animal model process were included in the study. Control groups were treated identically to the experimental groups, with standardised housing, handling, and testing conditions to minimise potential bias. Female mice were randomly allocated to the groups and the experimenter was blinded to the group assignments during behavioural testing and data analysis. The sample size in this study was determined using the resource equation method, with the minimum number of animals calculated as 12 and the maximum as 22 (Arifin and Zahiruddin, 2017). A total sample size of 16, consisting of 8 PNE subjects modelling ADHD and 8 control subjects generated under the same experimental conditions, was deemed acceptable. This sample size provided sufficient statistical power for the planned comparisons while adhering to ethical standards for the use of laboratory animals. All animal experimental protocols were reviewed and approved by the Animal Experimentation Ethics Committee at Hacettepe University (2020/08–06). Additionally, all procedures involving animals were conducted following the guidelines for the humane treatment of animals, ensuring minimal pain and distress while adhering to best practices in animal care and welfare.

### Behavioural analyses

To assess all three core symptoms of ADHD, specifically hyperactivity, attentional impairment, and impulsivity, we performed locomotor activity, Y-maze, and cliff avoidance tests during the fifth postnatal week. The open field test (OFT) was performed to evaluate anxiety-like behaviours, as anxiety is a common accompanying symptom of ADHD.

As it is estimated that 10 to 40% of ADHD-related changes are explained by environmental factors (Sciberras *et al*., 2017), we did not expect all offspring exposed to nicotine perinatally to develop ADHD-like behaviours. The Y-maze test was chosen to assess deficits in attentional performance and working memory, functions associated with the PFC (Kraeuter *et al*., 2019). Since inattention is a core and persistent symptom of ADHD linked to PFC dysfunction, this test served as a reliable measure for identifying ADHD-like cognitive impairments in PNE mice. Therefore, we selected the female and male pups showing decreased spontaneous alternation in the Y-maze test (an indicator of impaired attention) on postnatal week 5 and continued our analyses with those mice (for each sex-group combination, the sample size (n) was 4, Fig. 1B). This selection was based on the observation that inattentive symptoms tend to persist longer than hyperactive or impulsive behaviours, reflecting a more stable pattern in the manifestation of ADHD (Caye *et al*., 2016). Among all 17 pups perinatally exposed to nicotine, 8 (47.1%) were selected based on the above criteria (Fig. 1B). Eight mice, born from the female mice exposed to drinking water containing only sucrose, were included in the study as controls. These tests were chosen because ADHD symptoms are more likely to be associated with subregions of the PFC and related circuits (Yu *et al*., 2023).

### Locomotor activity and open field tests

OFT was performed to assess both locomotor activity (LMA) and anxiety-like behaviour. Each mouse was placed in a 22.5 × 22.5 × 30 cm arena and allowed to explore the environment for 10 minutes during which they were recorded with a video camera. The total distance travelled in the arena over a 10-minute period was used as a measure of LMA. The total time spent in the central 5×5 cm^2^ area of the arena during the first 5 minutes was used to assess anxiety-like behaviours. Ethovision XT-8 software was used for the analyses.

### Y-maze test

The Y-maze test (YMT) was used to evaluate prefrontal functions such as spatial working memory and attention that may be related to dorsal PFC. The maze consisted of three arms branching out from a triangular centre in a Y shape (each arm was 35 cm long × 6 cm wide × 10 cm high). Distinct visual cues were placed on the walls of all arms and the testing room to enable the mouse to uniquely recognise each arm. The behavioural task began with the mouse being placed at the centre of the Y-maze, and free access to all three arms was provided. The mouse’s behaviour was recorded using a video camera for 10 minutes. Video recordings were analysed to calculate the number of entries into each arm and the sequence of entries into the arms (for this purpose the arms were labelled A, B, and C). Entry into an arm was considered when all four limbs of the mouse entered that arm. A “spontaneous alternation” was defined as entry into three consecutive arms without repeated entry (e.g., ABC, BCA, CBA). A spontaneous alternation score was calculated using the formula: # alternations ÷ (# of entries - 2) × 100. The score correlates with spatial working memory and attention performance in the same direction.

### Cliff avoidance reaction test

A cliff avoidance reaction (CAR) test was performed to assess impulsivity that may be related to ventral PFC. The apparatus consisted of a specially designed round platform (20 cm in diameter) supported on iron legs (50 cm in height) (Yamashita *et al*., 2013). The test was initiated by placing the mouse in the centre of the platform. The mouse’s behaviour was recorded for 30 minutes using a video camera placed on the platform. Time spent in the centre 1/3 of the platform was calculated. Calculations regarding video recordings were made using Ethovision XT-8 software. Time spent in this area was considered as inversely related to impulsivity.

### Cortical thickness assessments

CTs of subregions of the PFC were measured to assess cortical maturation. Images were captured at 1x magnification using a Nikon Eclipse E600 fluorescence microscope from sections obtained from the mice’s PFCs. According to the mouse brain atlas, the regions selected based on stereotaxic coordinates are as follows: the ACC (1.10 to 0.02 mm), the PL (2.80 to 2.46 mm), and the IL (1.98 to 1.54 mm) (Franklin and Paxinos, 2008). The CTs of the ACC, PL, and IL were calculated from the obtained images using Image J software (Fig. 1C).

### Immunofluorescence staining

Inflammatory signalling pathways in the ACC, PL, and IL were investigated using immunohistochemical (IHC) labelling in PNE and control groups. After behavioural testing was completed, mice under anaesthesia were perfused with 1X phosphate-buffered saline (PBS) and 4% paraformaldehyde (PFA). After stripping the brains from the skull and post-fixed in 4% PFA solution for 24 h. The brains were then dehydrated with a 30% sucrose solution until they sank to the bottom of the solution. Coronal sections (20 μm) were serially cut on a Leica cryostat. Brain slices were selected from continuous frozen sections for immunofluorescence staining. Our study focused on labelling Iba-1, as well as the pro-inflammatory transcription factor NF-κB within the nucleus. Images were recorded using a Leica confocal microscope and processed with FIJI J software. The numbers of microglia labelled with Iba-1 were quantified. For the microglia marker Iba-1, Z-stacked images were also obtained, and the images were analysed using a MATLAB code, 3DMorph (York *et al*., 2018). To assess microglial morphology, we measured total cell volume and the ramification index, a quantitative measure of complexity and branching obtained through automated tracing of microglial processes in Z-stacks. The ramification index quantifies the extent to which a microglial projection branches, determined after applying thresholding, noise reduction, and skeletonisation to the projections. In contrast, the total cell volume represents the 3D volume of the entire microglia, including its processes, serving as an indirect measure of ramification. Observation of NF-κB signal within the nuclear area was interpreted as NF-κB activation. NF-κB activation was calculated as the ratio of the total number of cells with nuclear labelling of NF-κB to the total number of Hoechst-positive cells.

### Immunolabelling for Iba-1

After washing with 1X PBS, brain sections were permeabilised with 0.5% Triton X in PBS (PBST) for 15 minutes, blocked with 10% normal goat serum (NGS) in PBST for 1 hour at room temperature, and then incubated with Iba-1 (1:200) for 2 days at 4°C. Afterward, sections were washed with 1X PBS for 5 minutes three times and then incubated with 488/594 anti-rabbit antibody (1:200) at room temperature for 1 hour. Following this incubation, the sections were washed with 1X PBST for 15 minutes, and Hoechst stain was added to visualise the nuclei.

### *Immunolabelling for NF-*κ*B*

After washing with 1X PBS, brain sections were incubated in 10% citrate buffer at 80°C for 15 minutes. Following this incubation, sections were washed with 1X PBS for 5 minutes three times, permeabilised with 1X PBST for 15 minutes, blocked with 10% NGS in PBST for 1 hour at room temperature, and then incubated with NF-κB (1:200) for 2 days at 4°C. The remaining procedure was the same as immunolabelling with Iba-1.

### Statistical analysis

Statistical analyses for our study were conducted using SPSS software version 25.0. Results of PNE and control groups are presented as mean with standard error of the mean (SEM). A two-way analysis of variance (ANOVA) was utilised to assess statistical variances across sexes and groups. Mann Whitney *U* test was employed to conduct pairwise comparisons between PNE and control mice, categorised by sex, with the presentation of the median with interquartile range (IQR). Data regarding microglial morphology were assessed using one-way ANOVA with Bonferroni correction. A significance threshold of *P* < 0.05 was used to determine statistical significance. The graphics were made using Prism software version 8.0.

## Results

### Behavioural results

#### PNE mice had lower spontaneous alternation scores than controls

**Figure 2.**
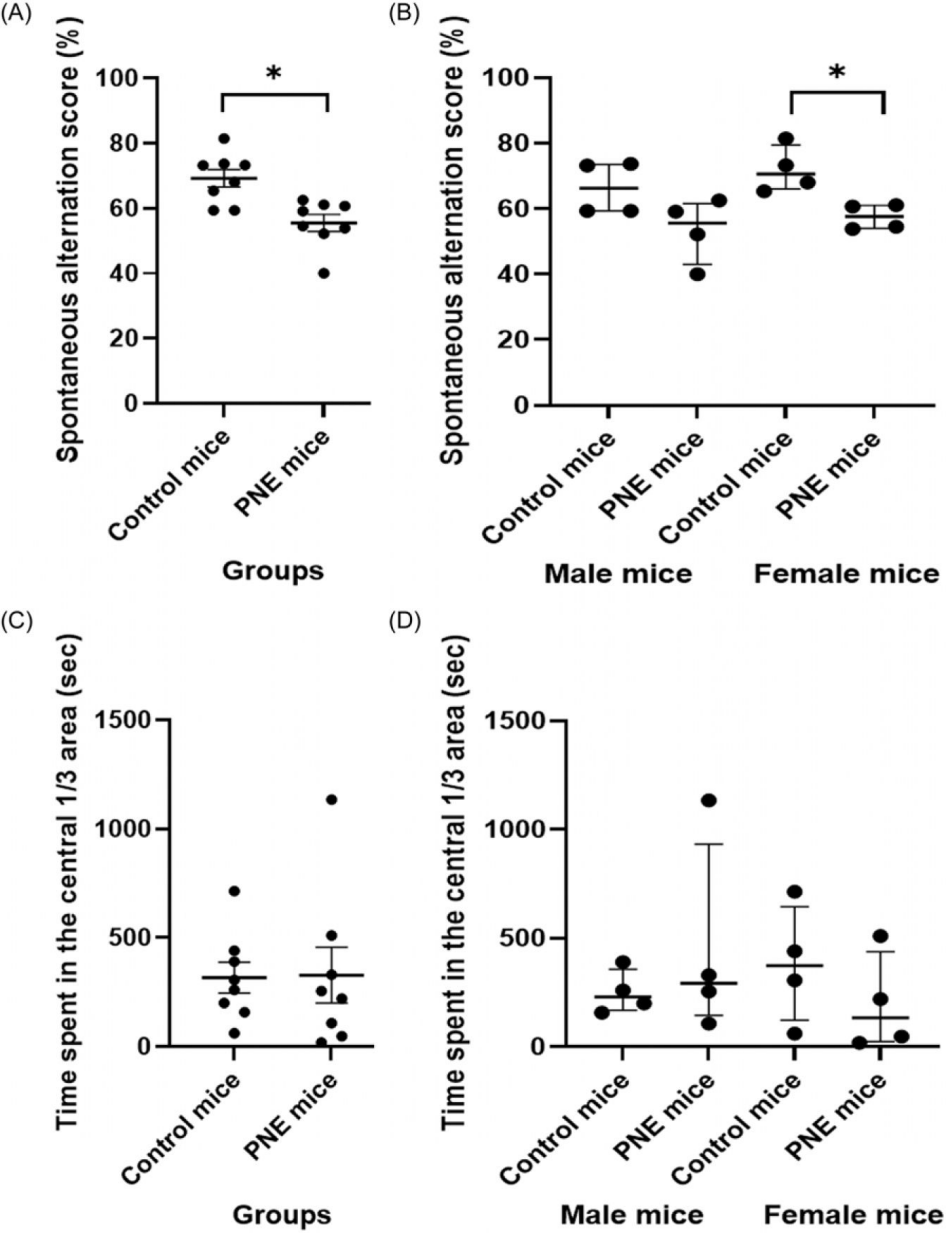
Behavioural performance in *Y*-maze and cliff avoidance reaction tests. (A) spontaneous alternation score (SAS) in the Y-maze test by group (%), shown as mean *±* SEM. (B) SAS by sex (%), shown as median *±* IQR. (C) time in central 1/3 area in the cliff avoidance reaction test by group (sec), shown as mean *±* SEM. (D) time in central 1/3 area by sex (sec), shown as median *±* IQR. *Note:* (*) indicates p-value<0.05.

Spontaneous alternation scores in YMT was significantly lower in PNE mice compared to control mice regardless of the sex (Fig. 2A and B, *F* (1,15) = 13.092, *p* = 0.004). There was no effect of sex on spontaneous alternation scores (*F* (1,15) = 1.656, *p* = 0.222). Specifically, PNE female mice exhibited fewer spontaneous alternations in YMT compared to control female mice (Fig. 2B, z = −2.309, *p* = 0.021). These findings confirmed that the selected PNE mice showed disturbances in spatial working memory and attention, functions mediated by PFC.

#### Cliff avoidance was similar across groups and sexes

The time spent in the central 1/3 area of the platform in the CAR test was similar between the PNE group and control mice (Fig. 2C and D, *F* (1,15) = 0.006, *p* = 0.939). Sex had no effect on the time spent in the central area of the platform (*F* (1,15) = 0.192, *p* = 0.669).

#### Locomotor activity was similar across groups and sexes, whereas anxiety-like behaviour shows group-specific changes but not sex-specific

**Figure 3.**
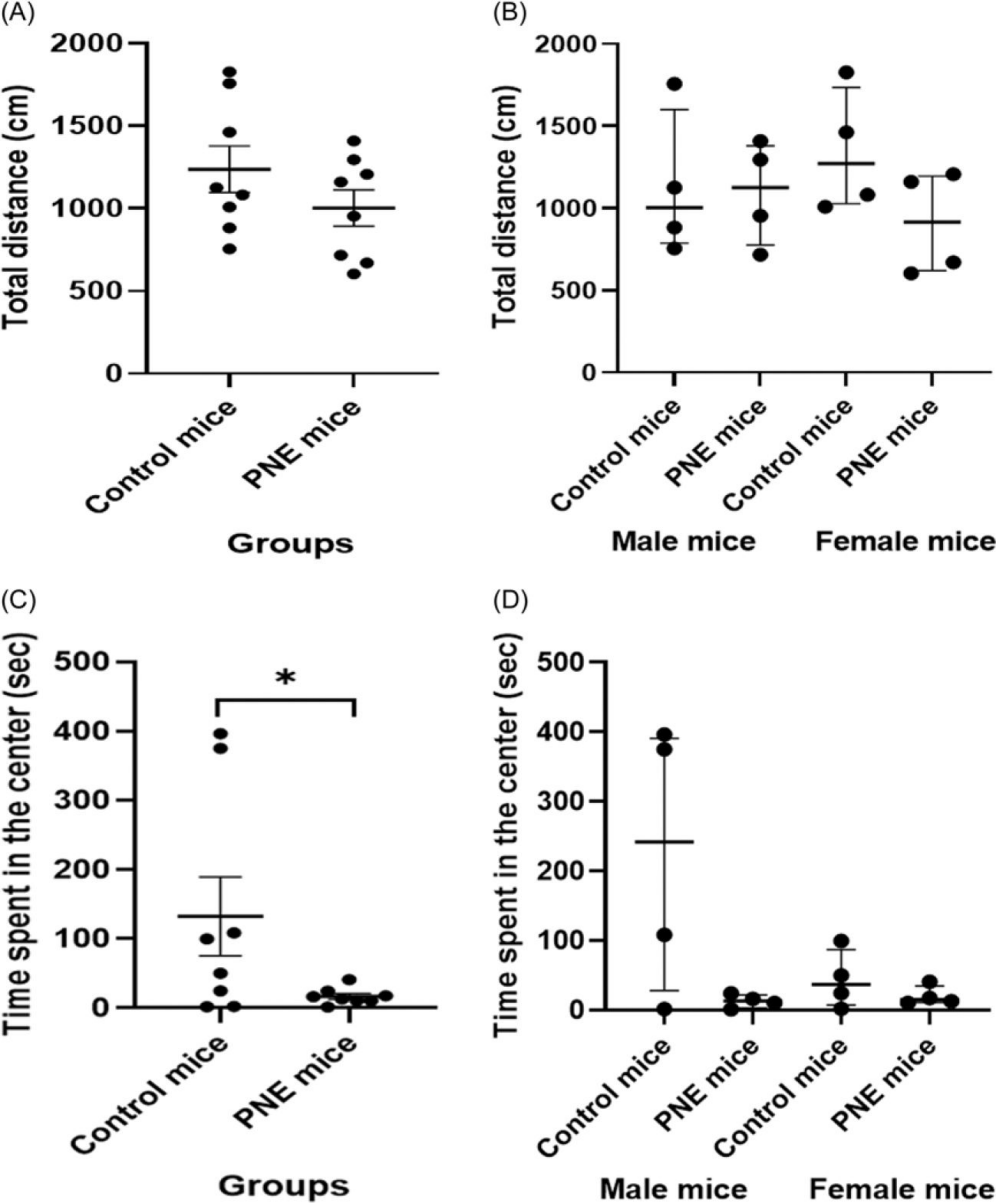
Behavioural performance in open field test. (A) locomotor activity (LMA) level by group (cm), shown as mean *±* SEM. (B) LMA by sex (cm), shown as median *±* IQR. (C) time spent at the centre in the open field test (sec) by group (sec), shown as mean *±* SEM. (D) time spent at the centre by sex (sec), shown as median *±* IQR. *Note:* (* ) indicates p-value<0.05.

When comparing LMA, the total distance traveled was similar between PNE group and control mice (Fig. 3A and B, *F* (1,15) = 1.632, *p* = 0.226). LMA findings were also similar across sexes (*F* (1,15) = 0.007, *p* = 0.933). These data indicate that the PNE group did not demonstrate increase in locomotor activity.

The PNE group spent less time in the central area in OFT, an indicator of anxiety-like behaviour, compared to the control group, regardless of the sex (Fig. 3C and D, *F* (1,15) = 5.274, *p* = 0.040). There was no effect of sex on time spent in the central area (*F* (1,15) = 2.820, *p* = 0.119). These findings indicate increased levels of anxiety-like behaviour in PNE mice in both sexes.

In summary, PNE mice demonstrated impairments in attention and working memory, but they didn’t display hyperactivity or increased impulsivity. Altogether these data suggest that PNE models the inattentive presentation of ADHD.

### Cortical thickness of dorsal PFC was increased in PNE mice

**Figure 4.**
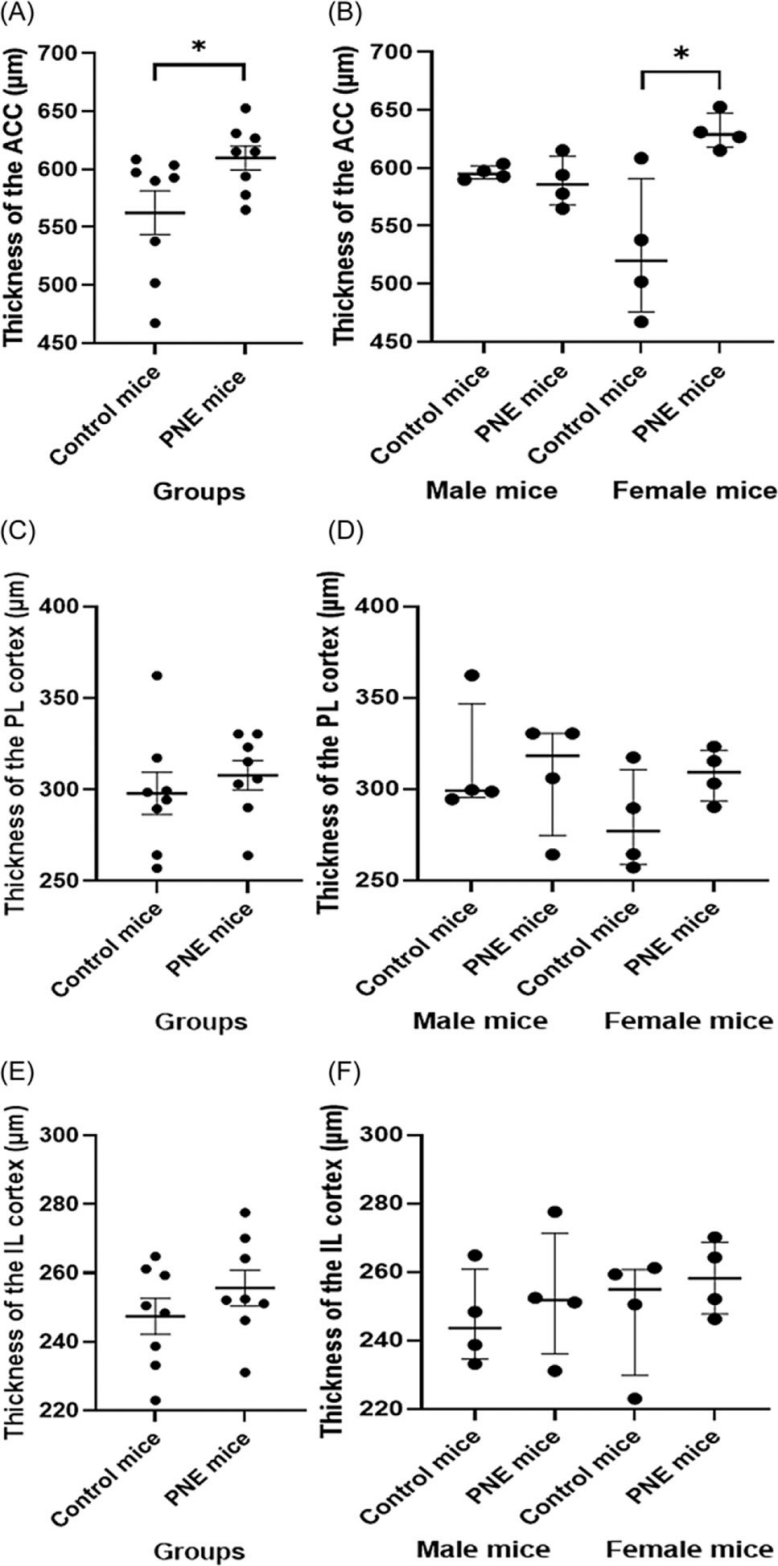
Cortical thickness measurements in prefrontal cortex subregions. (A) thickness of the anterior cingulate cortex (ACC) by group (μm), shown as mean *±* SEM. (B) ACC thickness by sex (μm), shown as median *±* IQR. (C) thickness of the prelimbic cortex (PL) by group (μm), shown as mean *±* SEM. (D) PL thickness by sex (μm), shown as median *±* IQR. (E) thickness of the infralimbic cortex (IL) by group (μm), shown as mean *±* SEM. (F) IL thickness by sex (μm), shown as median *±* IQR. *Note:* (*) indicates p-value<0.05.

We measured the thickness of all subdivisions of PFC, specifically of ACC, PL, IL across the cortical laminae. The ACC of PNE mice was thicker than that of the control mice (Fig. 4A and B, *F* (1,15) = 8.156, *p* = 0.014). While sex had no effect on the thickness of the ACC (*F* (1,15) = 0.504, *p* = 0.491), the sex–PNE interaction was significant (sex–group interaction: *F* (1,15) = 11.091, *p* = 0.006). Specifically, ACC CT was higher in PNE female mice when compared to that of control female mice (Fig. 4B, z = −2.309, *p* = 0.021).

Neither sex nor group had any effect on PL thickness (Fig. 4C and D, *F* (1,15) = 1.322, *p* = 0.273; and *F* (1,15) = 0.526, *p* = 0.482, respectively). Similarly, IL thickness was unaffected by either sex or group (Fig. 4E and F; *F* (1,15) = 0.217, *p* = 0.650; and *F* (1,15) = 1.098, *p* = 0.315, respectively).

### PNE groups of both sexes demonstrated higher microglial and NF-κB activation in medial prefrontal cortical areas

**Figure 5.**
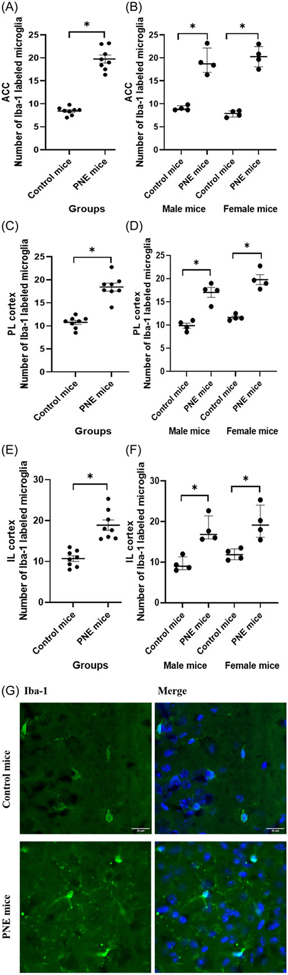
Microglia counts labelled with iba-1 in prefrontal cortex subregions. (A) number of microglia in the anterior cingulate cortex (ACC) by group, shown as mean *±* SEM. (B) ACC microglia count by sex, shown as median *±* IQR. (C) number of microglia in the prelimbic cortex (PL) by group, shown as mean *±* SEM. (D) PL microglia count by sex, shown as median *±* IQR. (E) Number of microglia in the infralimbic cortex (IL) by group, shown as mean *±* SEM. (F) IL microglia count by sex, shown as median *±* IQR. (G) Iba-1 labelling of microglia in the prefrontal cortex. *Note:* (*) Indicates p-value<0.05.

**Figure 6.**
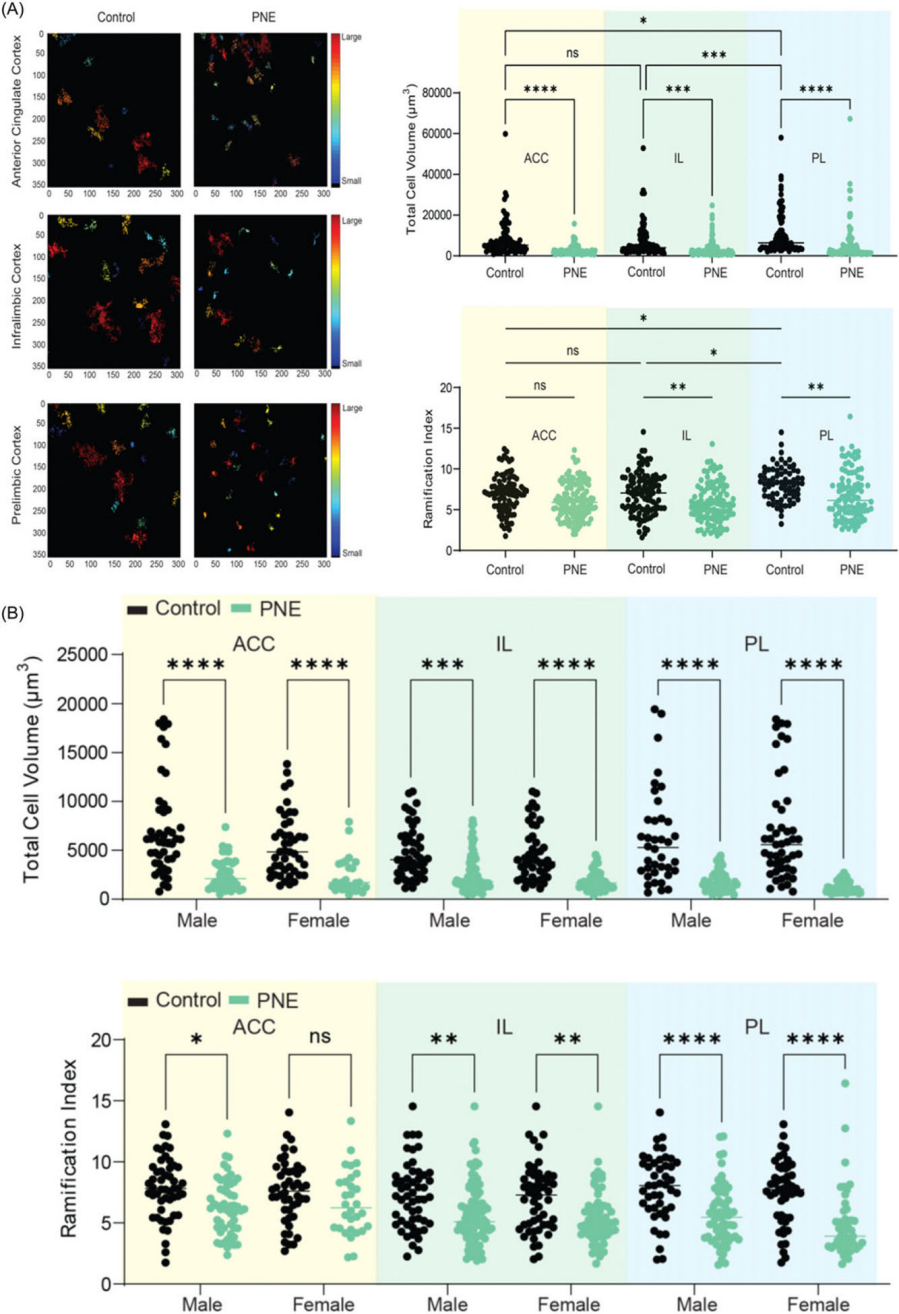
Microglial morphology in prefrontal cortex subregions. (A) data on ramification index and total cell volume in the anterior cingulate cortex (ACC), prelimbic (PL), and infralimbic (IL) cortices of PNE and control mice. (B) microglial morphology data by sex across ACC, PL, and IL cortices. *Note:* (*) indicates p-value<0.05.

PNE mice displayed higher numbers of Iba-1-labelled microglia in the ACC, PL, and IL than that control mice (Fig. 5A-G, *F* (1,15) = 143.257, *p* < 0.001; *F* (1,15) = 87.824, *p* < 0.001; and *F* (1,15) = 32.413, *p* < 0.001, respectively). Sex had no effect on the number of Iba-1-labelled microglia in the ACC and IL (*F* (1,15) = 0.009, *p* = 0.928; and *F* (1,15) = 2.125, *p* = 0.171, respectively). However, the number of Iba-1-labelled microglia in the PL of female mice was higher than that of male mice (*F* (1,15) = 7.564, *p* = 0.018). When comparing PNE and control groups separately for each sex, significant differences were observed in both PNE male and female mice across the ACC (Fig. 5B, z = −2.323, *p* = 0.020; and z = −2.309, *p* = 0.021, respectively), PL (Fig. 5D, z = −2.309, *p* = 0.021; and z = −2.323, *p* = 0.020, respectively), and IL (Fig. 5F, z = −2.309, *p* = 0.021; and z = −2.309, *p* = 0.021, respectively), compared to control male and female mice. Our analysis of microglial morphology revealed a significant reduction in total microglial volume across all three PFC subregions in PNE mice, indicative of microglial activation (Fig. 6A, all *p*’*s* < 0.001). Specifically, PNE mice demonstrated significantly lower ramification indices in the PL and IL cortices (*p* = 0.004 and *p* = 0.009, respectively), while microglial branching in the ACC remained comparable to that of control mice (Fig. 6A, *p* = 0.243). Notably, similar significant findings were also observed when comparing PNE and control groups within each sex (Fig. 6B, all *p*’*s* < 0.05). Moreover, we showed that resident microglia in the PL of the control group were significantly more ramified compared to those in the ACC and IL (Fig. 6A, *p* = 0.019 and *p* = 0.028, respectively). This may reflect regional differences in both the resting and activation states of microglia.

**Figure 7.**
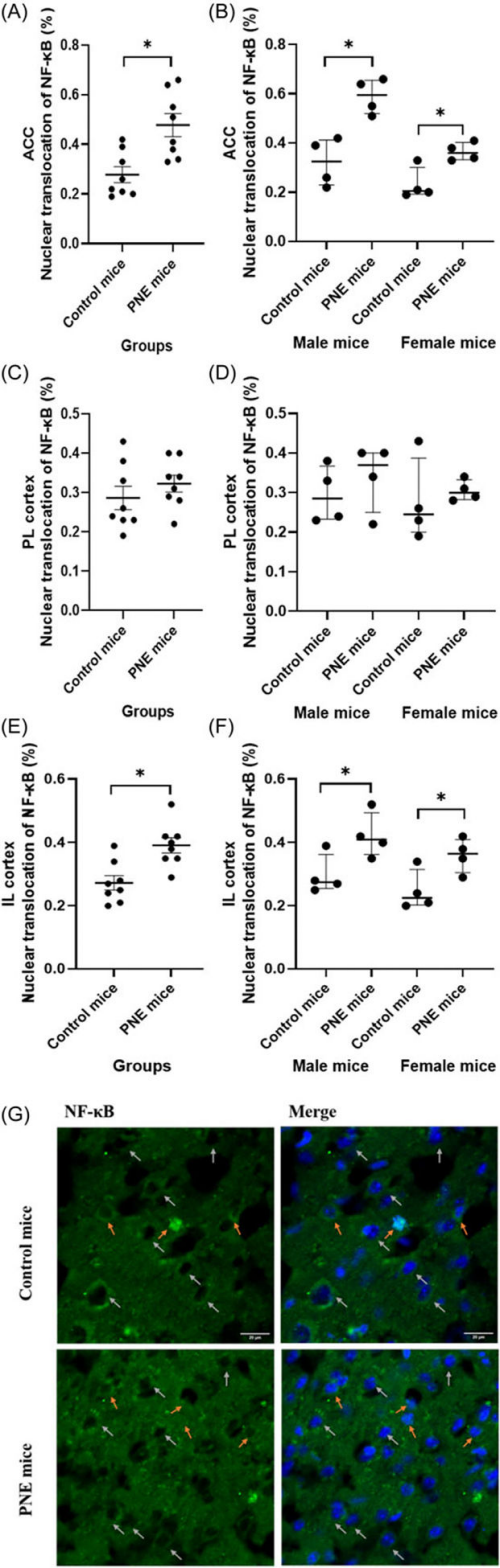
NF-κB nuclear translocation in prefrontal cortex subregions. (A) ratio of nuclear NF-κB-labelled cells the anterior cingulate cortex (ACC) by group (%), shown as mean *±* SEM. (B) ACC ratio by sex (%), shown as median *±* IQR. (C) ratio of nuclear NF-κB-labelled cells in the prelimbic cortex (PL) by group (%), shown as mean *±* SEM. (D) PL ratio by sex (%), shown as median *±* IQR. (E) ratio of nuclear NF-κB-labelled cells in the infralimbic cortex (IL) by group (%), shown as mean *±* SEM. (F) IL ratio by sex (%), shown as median *±* IQR. (G) NF-κB nuclear labeling in the prefrontal cortex. *Note:* (*) indicates p-value<0.05.

Nuclear translocation (i.e activation) of NF-κB was increased in the ACC and IL of PNE mice compared to controls (Fig. 7A-G, *F* (1,15) = 31.175, *p* < 0.001; and *F* (1,15) = 14.062, *p* = 0.003, respectively), in contrast to the PL (*F* (1,15) = 1.002, *p* = 0.337). The effect of sex on the ratio of nuclear translocated NF-κB cells in the ACC was significant, male mice had higher nuclear NF-κB ratios (*F* (1,15) = 19.030, *p* = 0.001). When comparing PNE and control groups separately for each sex, significant differences were observed in both PNE male and female mice across the ACC (Fig. 7B, z = −2.309, *p* = 0.021; and z = −2.309, *p* = 0.021, respectively) and IL (Fig. 7F, z = −2.021, *p* = 0.043; and z = −2.021, *p* = 0.043, respectively), compared to control male and female mice.

## Discussion

We evaluated neuroinflammatory changes in a PNE mouse model at 5th postnatal week, which corresponds to early adolescence. We observed that only 47.1% of the PNE mice developed ADHD-like attention impairments. PNE mice exhibited no differences in LMA and impulsivity but displayed a significant increase in anxiety-like behaviours when compared to controls. Our data is consistent with the inattentive presentation of ADHD, which is prevalent in both girls and boys.

Numerous cohort and meta-analysis studies link prenatal maternal smoking to a higher prevalence of ADHD in children (Huang *et al*., 2018). While several chemicals in cigarette smoke could mediate this effect, animal studies consistently indicate nicotine as the primary agent (Balsevich *et al*., 2014; Polli and Kohlmeier, 2020). Nicotine crosses the placenta, accumulates in fetal tissues, and can activate neuronal acetylcholine receptors, potentially disrupting progenitor cell signalling and leading to ADHD-like behaviours postnatally (Polli and Kohlmeier, 2020). Moreover, maternal smoking during pregnancy may activate the NF-κB pathway through the mediation of free radicals (Chen *et al.*, 2019). This can also lead to the activation of microglia, resulting in the overproduction of various pro-inflammatory cytokines and an enhanced local oxidative stress, likely mediated by toll-like receptors. In the brain, this exposure can increase levels of several pro-inflammatory cytokines, including tumour necrosis factor-α, interleukin (IL)-1 α, IL-1β, and IL-6 (Jones *et al*., 2020). Potential epigenetic modifications may contribute to the persistent upregulation of pro-inflammatory cytokine expression in the offspring’s brain. Similarly, PNE may contribute to the development of ADHD through neuroinflammatory processes. In our study, consistent with previous findings, a model of ADHD was established with PNE. However, no significant difference was observed in LMA and impulsivity compared to controls. In reviewing the literature, LMA in offspring following PNE may vary, being higher, lower, or similar compared to controls. LMA results are affected by factors such as sex, dosage, and timing of nicotine administration, and the age at which the test was performed (Pauly *et al*., 2004; Heath *et al*., 2010; Zhu *et al*., 2012; Alkam *et al*., 2013; Balsevich *et al*., 2014; Zhu *et al*., 2017; Zhang *et al*., 2018; Buck *et al*., 2019). Considering the significant anxiety accompanying male mice modelling ADHD in our study, the inclusion of an open-field component in the test assessing hyperactivity may have restricted the mobility of PNE mice. Therefore, in future studies, we recommend the assessment of hyperactivity in an environment that resembles the natural habitat of mice (Zhu *et al*., 2012). In line with the LMA, we note that the possible contribution of anxiety-like behaviours to the CAR that we used to assess impulsivity cannot be ignored (Zhu *et al*., 2017). Using the same model, results regarding cliff avoidance were inconsistent; PNE male offspring showed less cliff avoidance behaviour (impulsive-like behaviour), whereas females had no differences (Zhu *et al*., 2017). Furthermore, PNE offspring spent significantly more time in the cliff aversion position in the CAR test, whereas female ones travelled less, were more immobile, and spent less time in the central area in OFT (Liu *et al*., 2020). To more accurately assess impulsivity without the confounding effects of increased anxiety, future studies would benefit from using a paradigm that specifically measures the inability to resist a behaviour, such as the go/no-go test (Loos *et al*., 2010).

Consistent with the behavioural findings, we found that the thickness of the ACC was significantly higher in PNE mice compared to control mice, with a significant sex*group interaction. Specifically, the ACC was thicker in female PNE mice than in male PNE mice. Structural imaging studies have revealed that children with ADHD exhibit relatively slow cortical thinning, particularly in attention-associated regions such as the ACC (Rommelse *et al*., 2017). Studies indicate a negative correlation between the rate of cortical thinning in the dorsomedial (DMPFC; homolog of the ACC in mice) and ventromedial (VMPFC; that of the PL and IL) prefrontal regions and the severity of hyperactivity and impulsivity (Shaw *et al*., 2011; Le Merre *et al*., 2021). Typical developmental processes that involve the refinement of neural connections, such as synaptic pruning, may be delayed in individuals with ADHD, leading to the observed variations in brain structure and function (von Arx *et al*., 2023). Our finding of the ACC thickness was consistent with delayed cortical thinning and maturation, and increased CT observed regionally in ADHD (Levman *et al*., 2022). However, most research on childhood-onset ADHD has consistently found a predominantly thinner cortex and reduced brain volumes across widespread brain regions (Shaw *et al*., 2018). During adolescence, there is a significant reorganisation of activity and structure in the PFC of mice, highlighting a non-linear trajectory of prefrontal development, which is associated with most cognitive abilities (Pöpplau *et al*., 2024). The discrepancies between our findings and those of other studies, which have different timings and areas of focus, may stem from the temporal differences in the development of the PFC relative to other brain regions. However, these variations actually indicate underlying issues related to delayed maturation and, consequently, the pruning processes. Unlike the ACC, we found similar thicknesses of the PL and IL. Deficits in hot executive functions such as motivational control and reward-related decision-making, which are neurodevelopmental milestones expected to mature relatively early, are explained by changes in networks including the ventral PFC (Rubia, 2018). It is possible that we did not observe differences in hyperactivity and impulsivity-like behaviours, which are known to decrease with age in ADHD patients, because the PL and IL may have completed their maturation, similar to controls (Caye *et al*., 2016). Our finding of no differences in PL and IL thickness between PNE and control groups further supports this explanation. Conversely, activation changes in networks predominantly involving the dorsal PFC in individuals with ADHD are associated with deficits in later maturing cold executive functions such as motor inhibition and sustained attention (Rubia, 2011). Thus, increased CT of the ACC and attention-related impairment in PNE mice may be related to delayed cortical maturation (delayed thinning and pruning). In contrast to our measurements calculated from slices taken from more anterior regions, including the PL and IL as well as the ACC, Zhu *et al*. (2012) found a significant decrease in cingulate cortex volume and cingulate cortex length (radial thickness) in PNE group mice (sacrificed on postnatal day 42), but no decrease in the width (height) of the cingulate cortex in coronal sections representing the entire rostrocaudal extent. They argued that the decrease in radial thickness of the ACC reflects a decrease in the number of cells, dendritic branches, or axon terminals (Zhu *et al*., 2012). However, other animal studies using the PNE mouse model have suggested that nicotine causes persistent changes in dendritic spine density (Muhammad *et al*., 2012; Mychasiuk *et al*., 2013; Jung *et al*., 2016). Developmental nicotine exposure was found to alter neuron morphology in various cortical areas, typically grey matter, leading to a significant increase in fractional anisotropy measured by diffusion tensor imaging. These changes were suggested to be associated with increases in dendritic spine density, dendritic arborisation, and complexity, which can be interpreted as indirect evidence of delayed maturation and pruning processes (Jung *et al*., 2016). Nevertheless, large-scale imaging studies in recent years have highlighted that structural changes associated with ADHD are significantly less pronounced than those previously reported (Samea *et al*., 2019). The Adolescent Brain Cognitive Development (ABCD) study also found no significant differences in CT changes previously reported in ADHD (Casey *et al*., 2018). There is still a need to evaluate cortical maturation at the molecular level along with regionally associated higher-order cognitive functions using more advanced techniques.

As microglia are known to refine the dendritic tree, hence the cortical volume, we investigated the numbers and activation patterns in microglia. Microglia number and activity were increased in all three PFC subregions in both sexes. Specifically, within the ACC, which may contribute to the attentional impairment in PNE mice, this increase coincided with increased CT and elevated NF-κB activation. Increased levels of Iba-1 in the PL and IL, along with elevated NF-κB activation in the IL, were observed in PNE mice, which may correspond with a significant increase in anxiety-like behaviours compared to controls. Unlike males, PNE females exhibited attention-related impairments compared to female controls, along with increased cortical thickness in the ACC.

Our findings align with studies showing increased proinflammatory cytokines and microglial activation in spontaneously hypertensive rats (SHRs), particularly elevated Iba-1 labelling in the PFC (K Tayebati *et al*., 2016; Fang *et al*., 2023). These results support the role of neuroinflammatory signalling and blood–brain barrier disruption in ADHD pathophysiology. Methylphenidate, a common ADHD treatment, reduced cognitive impairments and microglial numbers in the medial PFC (Ramon-Duaso *et al*., 2019), even reverting microglial morphology from active to resting states in SHRs (Coelho-Santos *et al*., 2018). Increased microglial activation has been linked to ADHD symptom severity, such as processing speed and attention deficits (Yokokura *et al*., 2021). Our research also indicated that NF-κB activation correlated with rising microglial numbers, particularly in the dorsal PFC. Alarmins enhance microglial migration and phagocytosis via the TLR4/NF-κB pathway (Zhang *et al*., 2023; Bai *et al*., 2023), which aligns with the NF-κB-microglia proliferation pathway proposed by Uzay *et al*. (2024). Maternal nicotine exposure impacts neurogenesis and microglial activity, affecting anxiety-like behaviours in offspring (Liu *et al*., 2020). Transient prefrontal microglia deficiency in adolescence results in a greater overall decrease in synaptic density on pyramidal neurons in the dorsal subregions of the PFC than in the ventral subregions, resulting in reduced cognitive abilities (Schalbetter *et al*., 2022). In adolescent PNE mice, we noted reduced microglial branching, indicating heightened activity, which may disrupt synapse formation and lead to cognitive impairments (von Arx *et al*., 2023). Overall, neuroinflammatory changes in the dorsal PFC appear to be associated with attention-related impairments.

The colonisation of microglia during embryonic development largely relies on blood vessels and circulation. The growth of microglia is affected by their brain location and distance from blood vessels, with more complex branching occurring in areas with better blood supply (Harry, 2013). PNE may decrease capillary blood flow in the fetal brain, which could delay the proper maturation of microglia into their ramified form and cause microglial hyperactivation in the offspring (Harry, 2013; Nakayama *et al*., 2019; Liu *et al*., 2020). During normal development, the activity of the medial PFC and cognitive abilities, follow a pattern of increase in adolescence (Schalbetter *et al*., 2022). This is accompanied by microglia-mediated structural changes, such as the remodelling of synaptic density and dendritic branching (Wang *et al*., 2022). In mice, early adolescence (around postnatal day 30) is marked by structural and functional reorganisation through microglia-mediated pruning (Pöpplau *et al*., 2024). Adolescent microglia have a more rounded and less branched morphology, indicating increased phagocytic activity. This process is crucial for the development of neural circuits and higher-order cognitive skills. However, if microglial dysfunction or hyperactivation occurs at this stage, it may alter prefrontal network activity and morphology, such as an increase or decrease in spine densities, potentially leading to cognitive impairments that persist into adulthood (Schalbetter *et al*., 2022; Pöpplau *et al*., 2024). In alignment with the literature, PNE mice exhibited more rounded and less branched microglia within the medial prefrontal areas, indicative of microglial hyperactivity. These changes were also accompanied by increased numbers of microglia and pro-inflammatory NF-κB activation, indicating the extent of neuroinflammation. Age-dependent microglia-mediated pruning during adolescence enables region-specific maturation, leading to the acquisition of various cognitive skills (von Arx *et al*., 2023). A similar hierarchy may exist among the subregions of the PFC (Teissier & Pierani, 2021). In the control group, we found that PL cortical resident microglia were significantly more ramified compared to those in the ACC and IL, indicating a resting state and interpreted that the PL may have matured earlier than other subregions. Thus, we did not observe any differences in CT in the PL and IL in adolescent PNE mice compared with controls, although this would have later progressed to a decrease in CT due to increased numbers and hyperactivity of microglia. Interestingly, in the ACC, which is likely to be the last subregion to mature, we observed increased CT and attentional impairment due to more branched and hence less active microglia compared to controls, suggesting delayed maturation in the dorsal PFC of PNE mice. Since synaptic pruning occurs earlier in females than in males during adolescence, the observed differences were also pronounced among females in both groups (von Arx *et al*., 2023).

We observed a pattern of increased NF-κB activation in the ACC and IL. The transcription factor NF-κB induces the expression and release of various proinflammatory genes, such as cytokines, chemokines, and adhesion molecules, as well as microglial phagocytosis, by regulating different aspects of neuroinflammatory signalling, neuroprotection, and apoptosis (Shabab *et al*., 2017). Human studies investigating the pathophysiology of neurodevelopmental disorders which also include autism spectrum disorder (ASD) have demonstrated inflammatory signalling involving NF-κB activation in peripheral blood samples as well as post-mortem brain tissues. Similarly, animal studies utilising ASD models have shown consistent evidence of neuroinflammation in brain tissues (Liao & Li, 2020). Levels of cytokines and chemokines related to the NF-κB pathway rather than direct NF-κB expression have been extensively investigated in peripheral blood samples of children and adolescents with ADHD. Low levels of inflammatory markers were detected in these studies (Misiak *et al*., 2022). A prospective study also showed that levels of NF-κB pathway-related cytokines in mothers’ peripheral blood samples during pregnancy predicted ADHD symptoms in children aged 4–6 years (Gustafsson *et al*., 2020). In light of all the evidence, prenatal risk factors such as maternal stress, obesity, and maternal smoking may trigger maternal inflammation during the prenatal period, leading to neuroinflammatory changes in children and thus neurodevelopmental problems such as ADHD, through a common end pathway. However, further studies investigating the genetic predisposition to impaired synaptic plasticity and the epigenetic mechanisms associated with environmental risk factors, such as synaptic pruning deficits, are needed to support these findings.

We included both male and female mice to better represent ADHD in our study. However, the sample size was relatively small and the findings, specifically those regarding sex differences need to be extended by future studies. While this model offers insights into the interplay between genetic and environmental factors through prenatal nicotine exposure, the high heritability and clinical heterogeneity of ADHD may limit the generalisability of our results. Moreover, variability was observed among PNE mice, with some exhibiting ADHD-like behaviours while others did not, indicating a possible role for genetic susceptibility in modulating the effects of this environmental factor. Future research could benefit from exploring this genetic predisposition more directly. The behavioural tests applied (Y-maze, LMA, open field and cliff avoidance tests) were chosen to assess a range of ADHD-relevant domains and PFC functions. However, while informative, they lack the specificity needed for a more precise evaluation of attention and impulse control. Expanding the behavioural test battery in future studies could provide further clarity. Finally, despite the small sample size, our examination of sex differences between male and female mice offers a potential foundation for future research.

## Conclusion

Overall, we observed increases in CT, microglial cell number, activity, and NF-κB activation in the ACC, which contribute to attention-related impairments in PNE mice. Our findings highlight the role of neuroinflammatory signalling in ADHD pathophysiology and may inform future treatment strategies targeting these mechanisms. Additionally, we noted minor differences in ADHD-like behaviours, CT, and neuroinflammatory signalling between PNE female and male mice. While these sex differences are not definitive due to small sample sizes, they suggest potential variations and underscore the need for future studies with larger cohorts to explore this further.

## Data Availability

The dataset used can be accessed by contacting the corresponding author.

## Significant outcomes

- Perinatal nicotine exposure (PNE) induces neuroinflammatory signalling, evidenced by increased cortical thickness, microglial activation, and NF-κB activation in the anterior cingulate cortex, contributing to attention-related impairments resembling the inattentive presentation of attention-deficit/hyperactivity disorder (ADHD).
- Anxiety-like behaviours, but not impulsivity or locomotor activity, are significantly elevated in PNE mice, correlating with neuroinflammatory changes in the prelimbic and infralimbic cortices, highlighting the interaction between inflammation and comorbid behaviours of ADHD.
- Observed sex differences in ADHD-like behaviours and neuroinflammatory markers, while limited by sample size, point to potential sex-specific mechanisms in ADHD pathophysiology, warranting further exploration in larger cohorts.

## Limitations

- The small sample size limits the statistical power and generalisability of findings, particularly in evaluating sex differences and behavioural heterogeneity.
- Behavioural tests focused on a subset of attention-deficit/hyperactivity disorder (ADHD) domains; additional assessments could provide deeper insights into impulsivity and attention deficits.
- While the mouse model offers insights into environmental exposures, the high heritability and clinical heterogeneity of ADHD in humans may reduce its direct applicability to broader populations.

## References

[R1] Alkam T, Kim H-C, Mamiya T, Yamada K, Hiramatsu M and Nabeshima T (2013) Evaluation of cognitive behaviors in young offspring of C57BL/6J mice after gestational nicotine exposure during different time-windows. Psychopharmacology 230(3), 451–463. 23793357 10.1007/s00213-013-3175-9

[R2] Anand D, Colpo GD, Zeni G, Zeni CP and Teixeira AL (2017) Attention-deficit/hyperactivity disorder and inflammation: what does current knowledge tell us? A systematic, review. Frontiers in Psychiatry 8, 228. 29170646 10.3389/fpsyt.2017.00228 PMC5684106

[R3] Arifin WN and Zahiruddin WM (2017) Sample size calculation in animal studies using the resource equation approach. The Malaysian Journal of Medical Sciences 24(5), 101–105. 10.21315/mjms2017.24.5.11 PMC5772820 29386977

[R4] Ayano G, Demelash S, Gizachew Y, Tsegay L and Alati R (2023) The global prevalence of attention deficit hyperactivity disorder in children and adolescents: An umbrella review of meta-analyses. Journal of Affective Disorders 339, 860–866. 37495084 10.1016/j.jad.2023.07.071

[R5] Bai Q, Sun D, Zeng Y, Zhu J, Zhang C, Zhang X, Chen L, Zhou X, Ye L and Tang Y (2023) Effect of proinflammatory S100A9 protein on migration and proliferation of microglial cells. Journal of Molecular Neuroscience 73(11–12), 983–995. 37947991 10.1007/s12031-023-02168-1

[R6] Balsevich G, Poon A, Goldowitz D and Wilking JA (2014) The effects of pre- and post-natal nicotine exposure and genetic background on the striatum and behavioral phenotypes in the mouse. Behavioural Brain Research 266, 7–18. 24607511 10.1016/j.bbr.2014.02.038

[R7] Bianchi ME (2007) DAMPs, PAMPs, and alarmins: all we need to know about danger. Journal of Leucocyte Biology 81(1), 1–5. 10.1189/jlb.0306164 17032697

[R8] Bryden DW, Burton AC, Barnett BR, Cohen VJ, Hearn TN, Jones EA, Kariyil RJ, Kunin A, In Kwak S and Lee J (2016) Prenatal nicotine exposure impairs executive control signals in medial prefrontal cortex. Neuropsychopharmacology 41(3), 716–725. 26189451 10.1038/npp.2015.197 PMC4707818

[R9] Buck JM, Sanders KN, Wageman CR, Knopik VS, Stitzel JA and O’Neill HC (2019) Developmental nicotine exposure precipitates multigenerational maternal transmission of nicotine preference and ADHD-like behavioral, rhythmometric, neuropharmacological, and epigenetic anomalies in adolescent mice. Neuropharmacology 149, 66–82. 30742847 10.1016/j.neuropharm.2019.02.006 PMC7096676

[R10] Carucci S, Narducci C, Bazzoni M, Balia C, Donno F, Gagliano A and Zuddas A (2023) Clinical characteristics, neuroimaging findings, and neuropsychological functioning in attention-deficit hyperactivity disorder: sex differences. Journal of Neuroscience Research 101(5), 704–717. 35293009 10.1002/jnr.25038

[R11] Casey BJ, Cannonier T, Conley MI, Cohen AO, Barch DM, Heitzeg MM, Soules ME, Teslovich T, Dellarco DV, Garavan H, Orr CA, Wager TD, Banich MT, Speer NK, Sutherland MT, Riedel MC, Dick AS, Bjork JM, Thomas KM, Chaarani B, Mejia MH, Hagler DJ, Daniela Cornejo M, Sicat CS, Harms MP, Dosenbach NUF, Rosenberg M, Earl E, Bartsch H, Watts R, Polimeni JR, Kuperman JM, Fair DA and Dale AM (2018) The adolescent brain cognitive development (ABCD) study: imaging acquisition across 21 sites. Developmental Cognitive Neuroscience 32, 43–54. 29567376 10.1016/j.dcn.2018.03.001 PMC5999559

[R12] Caye A, Swanson J, Thapar A, Sibley M, Arseneault L, Hechtman L, Arnold LE, Niclasen J, Moffitt T and Rohde LA (2016) Life span studies of ADHD—conceptual challenges and predictors of persistence and outcome. Current Psychiatry Reports 18(12), 1–11. 27783340 10.1007/s11920-016-0750-x PMC5919196

[R13] Cecil CA and Nigg JT (2022) Epigenetics and ADHD: reflections on current knowledge, research priorities, and translational potential. Molecular Diagnosis & Therapy 26(6), 581–606. 35933504 10.1007/s40291-022-00609-y PMC7613776

[R14] Chan YL, Oliver BG and Chen H (2020) What lessons have we learnt about the impact of maternal cigarette smoking from animal models? Clinical and Experimental Pharmacology and Physiology 47(2), 337–344. 31556137 10.1111/1440-1681.13182

[R15] Chan YL, Saad S, Pollock C, Oliver B, Al-Odat I, Zaky AA, Jones N and Chen H (2016) Impact of maternal cigarette smoke exposure on brain inflammation and oxidative stress in male mice offspring. Scientific Reports 6(1), 25881. 27169932 10.1038/srep25881 PMC4864383

[R16] Chen H, Chan YL, Oliver BG, Pollock CA, and Saad S (2019) Maternal smoking and fetal brain outcome: mechanisms and possible solutions. Neuroscience of Nicotine 9, 9–16.

[R17] Chuang Y-C, Wang C-Y, Huang W-L, Wang L-J, Kuo H-C, Chen Y-C and Huang Y-J (2022) Two meta-analyses of the association between atopic diseases and core symptoms of attention deficit hyperactivity disorder. Scientific Reports 12(1), 3377. 35232975 10.1038/s41598-022-07232-1 PMC8888762

[R18] Coelho-Santos V, Cardoso FL, Leitão RA, Fontes-Ribeiro CA and Silva AP (2018) Impact of developmental exposure to methylphenidate on rat brain’s immune privilege and behavior: control versus ADHD model. Brain, Behavior, and Immunity 68, 169–182. 29061363 10.1016/j.bbi.2017.10.016

[R19] Contreras D, Piña R, Carvallo C, Godoy F, Ugarte G, Zeise M, Rozas C and Morales B (2022) Methylphenidate restores behavioral and neuroplasticity impairments in the prenatal nicotine exposure mouse model of ADHD: evidence for involvement of AMPA receptor subunit composition and synaptic spine morphology in the hippocampus. International Journal of Molecular Sciences 23(13), 7099. 35806103 10.3390/ijms23137099 PMC9266648

[R20] Cortese S (2012) The neurobiology and genetics of attention-deficit/hyperactivity disorder (ADHD): what every clinician should know. European Journal of Paediatric Neurology 16(5), 422–433. 22306277 10.1016/j.ejpn.2012.01.009

[R21] Cowan M and Petri WA Jr. (2018) Microglia: immune regulators of neurodevelopment. Frontiers in Immunology 9, 2576. 30464763 10.3389/fimmu.2018.02576 PMC6234957

[R22] Fang Z, Shen G, Amin N, Lou C, Wang C and Fang M (2023) Effects of neuroinflammation and autophagy on the structure of the blood-brain barrier in ADHD model. Neuroscience 530, 17–25. 37625689 10.1016/j.neuroscience.2023.08.025

[R23] Faraone SV, Banaschewski T, Coghill D, Zheng Y, Biederman J, Bellgrove MA, Newcorn JH, Gignac M, Al Saud NM, Manor I, Rohde LA, Yang L, Cortese S, Almagor D, Stein MA, Albatti TH, Aljoudi HF, Alqahtani MMJ, Asherson P, Atwoli L, Bölte S, Buitelaar JK, Crunelle CL, Daley D, Dalsgaard Søren, Döpfner M, Espinet (on behalf of CADDRA) S, Fitzgerald M, Franke B, Gerlach M, Haavik J, Hartman CA, Hartung CM, Hinshaw SP, Hoekstra PJ, Hollis C, Kollins SH, Sandra Kooij JJ, Kuntsi J, Larsson H, Li T, Liu J, Merzon E, Mattingly G, Mattos P, McCarthy S, Mikami AY, Molina BSG, Nigg JT, Purper-Ouakil D, Omigbodun OO, Polanczyk GV, Pollak Y, Poulton AS, Rajkumar RP, Reding A, Reif A, Rubia K, Rucklidge J, Romanos M, Ramos-Quiroga JA, Schellekens A, Scheres A, Schoeman R, Schweitzer JB, Shah H, Solanto MV, Sonuga-Barke E, Soutullo César, Steinhausen H-C, Swanson JM, Thapar A, Tripp G, van de Glind G, van den Brink W, Van der Oord S, Venter A, Vitiello B, Walitza S and Wang Y (2021) The world federation of ADHD international consensus statement: 208 evidence-based conclusions about the disorder. Neuroscience & Biobehavioral Reviews 128, 789–818. 33549739 10.1016/j.neubiorev.2021.01.022 PMC8328933

[R24] Franklin KB and Paxinos G (2008) The Mouse Brain in Stereotaxic Coordinates. Amsterdam, Boston: Elsevier/Academic Press.

[R25] Giana G, Romano E, Porfirio MC, D’Ambrosio R, Giovinazzo S, Troianiello M, Barlocci E, Travaglini D, Granstrem O, Pascale E, Tarani L, Curatolo P, Laviola G, Adriani W (2015) Detection of auto-antibodies to DAT in the serum: interactions with DAT genotype and psycho-stimulant therapy for ADHD. Journal of Neuroimmunology 278, 212–222. 25468771 10.1016/j.jneuroim.2014.11.008

[R26] Gustafsson HC, Sullivan EL, Battison EA, Holton KF, Graham AM, Karalunas SL, Fair DA, Loftis JM and Nigg JT (2020) Evaluation of maternal inflammation as a marker of future offspring ADHD symptoms: a prospective investigation. Brain, Behavior, and Immunity 89, 350–356. 32707260 10.1016/j.bbi.2020.07.019 PMC7703804

[R27] Harry GJ (2013) Microglia during development and aging. Pharmacology & Therapeutics 139(3), 313–326. 23644076 10.1016/j.pharmthera.2013.04.013 PMC3737416

[R28] Havdahl A, Wootton RE, Leppert B, Riglin L, Ask H, Tesli M, Askeland RB, Hannigan LJ, Corfield E, Øyen A-S, Andreassen OA, Tilling K, Smith GD, Thapar A, Reichborn-Kjennerud T and Stergiakouli E (2022) Associations between pregnancy-related predisposing factors for offspring neurodevelopmental conditions and parental genetic liability to attention-deficit/hyperactivity disorder, autism, and schizophrenia: the norwegian mother, father and child cohort study (MoBa). JAMA Psychiatry 79(8), 799–810. 35793100 10.1001/jamapsychiatry.2022.1728 PMC9260642

[R29] Heath CJ, Horst NK and Picciotto MR (2010) Oral nicotine consumption does not affect maternal care or early development in mice but results in modest hyperactivity in adolescence. Physiology & Behavior 101(5), 764–769. 20826170 10.1016/j.physbeh.2010.08.021 PMC2975773

[R30] Huang L, Wang Y, Zhang L, Zheng Z, Zhu T, Qu Y and Mu D (2018) Maternal smoking and attention-deficit/hyperactivity disorder in offspring: a meta-analysis. Pediatrics 141(1), e20172465. 10.1542/peds.2017-2465 29288161

[R31] Jones DR, Smyth JM, Engeland CG, Sliwinski MJ, Russell MA, Sin NL and Graham-Engeland JE (2020) Affect variability and inflammatory markers in midlife adults. Health Psychology 39(8), 655–666. 32324001 10.1037/hea0000868 PMC8351733

[R32] Jung Y, Hsieh LS, Lee AM, Zhou Z, Coman D, Heath CJ, Hyder F, Mineur YS, Yuan Q and Goldman D (2016) An epigenetic mechanism mediates developmental nicotine effects on neuronal structure and behavior. Nature Neuroscience 19(7), 905–914. 27239938 10.1038/nn.4315 PMC4925298

[R33] Kim JH, Kim JY, Lee J, Jeong GH, Lee E, Lee S, Lee KH, Kronbichler A, Stubbs B and Solmi M (2020) Environmental risk factors, protective factors, and peripheral biomarkers for ADHD: An umbrella review. The Lancet Psychiatry 7(11), 955–970. 33069318 10.1016/S2215-0366(20)30312-6

[R34] Kozłowska A, Wojtacha P, Równiak M, Kolenkiewicz M and Huang ACW (2019) ADHD pathogenesis in the immune, endocrine, and nervous systems of juvenile and maturing SHR and WKY rats. Psychopharmacology 236(10), 2937–2958. 30737597 10.1007/s00213-019-5180-0 PMC6820808

[R35] Kraeuter AK, Guest PC and Sarnyai Z (2019) The Y-maze for assessment of spatial working and reference memory in mice. Methods in Molecular Biology 1916, 105–111. 30535688 10.1007/978-1-4939-8994-2_10

[R36] Krone B, Newcorn JH (2015) Comorbidity of ADHD and anxiety disorders. In Adler LA, Spencer TJ and Wilens TE(eds), Attention-eficit Hyperactivity Disorder in Adults and Children. Cambridge University Press, pp. 98–110.

[R37] Le Merre P, Ährlund-Richter S and Carlén M (2021) The mouse prefrontal cortex: unity in diversity. Neuron 109(12), 1925–1944. 33894133 10.1016/j.neuron.2021.03.035

[R38] Levman J, Forgeron C, Shiohama T, MacDonald P, Stewart N, Lim A, Berrigan L and Takahashi E (2022) Cortical thickness abnormalities in attention deficit hyperactivity disorder revealed by structural magnetic resonance imaging: newborns to young adults. International Journal of Developmental Neuroscience 82(7), 584–595. 35797727 10.1002/jdn.10211

[R39] Liao X and Li Y (2020) Nuclear factor kappa B in autism spectrum disorder: a systematic review. Pharmacological Research 159, 104918. 10.1016/j.phrs.2020.104918 32461184

[R40] Liu F, Tao X, Pang G, Wu D, Hu Y, Xue S, Liu J, Li B, Zhou L and Liu Q (2020) Maternal nicotine exposure during gestation and lactation period affects behavior and hippocampal neurogenesis in mouse offspring. Frontiers in Pharmacology 10, 1569. 32038246 10.3389/fphar.2019.01569 PMC6987079

[R41] Loos M, Staal J, Schoffelmeer AN, Smit AB, Spijker S and Pattij T (2010) Inhibitory control and response latency differences between C57BL/6J and DBA/2J mice in a Go/No-Go and 5-choice serial reaction time task and strain-specific responsivity to amphetamine. Behavioural Brain Research 214(2), 216–224. 20580749 10.1016/j.bbr.2010.05.027

[R42] Misiak B, Wojta-Kempa M, Samochowiec J, Schiweck C, Aichholzer M, Reif A, Samochowiec A and Stańczykiewicz B (2022) Peripheral blood inflammatory markers in patients with attention deficit/hyperactivity disorder (ADHD): a systematic review and meta-analysis. Progress in Neuro-Psychopharmacology and Biological Psychiatry 118, 110581. 10.1016/j.pnpbp.2022.110581 35660454

[R43] Mordelt A and de Witte LD (2023) Microglia-mediated synaptic pruning as a key deficit in neurodevelopmental disorders: hype or hope? Current Opinion in Neurobiology 79, 102674. 10.1016/j.conb.2022.102674 36657237

[R44] Muhammad A, Mychasiuk R, Nakahashi A, Hossain SR, Gibb R and Kolb B (2012) Prenatal nicotine exposure alters neuroanatomical organization of the developing brain. Synapse 66(11), 950–954. 22837140 10.1002/syn.21589

[R45] Mychasiuk R, Muhammad A, Gibb R and Kolb B (2013) Long-term alterations to dendritic morphology and spine density associated with prenatal exposure to nicotine. Brain Research 1499, 53–60. 23328078 10.1016/j.brainres.2012.12.021

[R46] Nakayama A, Yoshida M, Kagawa N and Nagao T (2019) The neonicotinoids acetamiprid and imidacloprid impair neurogenesis and alter the microglial profile in the hippocampal dentate gyrus of mouse neonates. Journal of Applied Toxicology 39(6), 877–887. 30693975 10.1002/jat.3776

[R47] Pauly JR, Sparks JA, Hauser KF and Pauly TH (2004) In utero nicotine exposure causes persistent, gender-dependent changes in locomotor activity and sensitivity to nicotine in C57Bl/6 mice. International Journal of Developmental Neuroscience 22(5–6), 329–337. 15380832 10.1016/j.ijdevneu.2004.05.009

[R48] Polli FS and Kohlmeier KA (2018) Prenatal nicotine exposure alters postsynaptic AMPA receptors and glutamate neurotransmission within the laterodorsal tegmentum (LDT) of juvenile mice. Neuropharmacology 137, 71–85. 29751228 10.1016/j.neuropharm.2018.04.024

[R49] Polli FS and Kohlmeier KA (2020) Prenatal nicotine exposure in rodents: why are there so many variations in behavioral outcomes? Nicotine and Tobacco Research 22(10), 1694–1710. 31595949 10.1093/ntr/ntz196

[R50] Polli FS, Scharff MB, Ipsen TH, Aznar S, Kohlmeier KA and Andreasen JT (2020) Prenatal nicotine exposure in mice induces sex-dependent anxiety-like behavior, cognitive deficits, hyperactivity, and changes in the expression of glutamate receptor associated-genes in the prefrontal cortex. Pharmacology Biochemistry and Behavior 195, 172951. 10.1016/j.pbb.2020.172951 32439454

[R51] Pöpplau JA, Schwarze T, Dorofeikova M, Pochinok I, Günther A, Marquardt A and Hanganu-Opatz IL (2024) Reorganization of adolescent prefrontal cortex circuitry is required for mouse cognitive maturation. Neuron 112(3), 421–440.e7. 37979584 10.1016/j.neuron.2023.10.024 PMC10855252

[R52] Ramon-Duaso C, Gener T, Consegal M, Fernández-Avilés C, Gallego JJé, Castarlenas L, Swanson MS, de la Torre R, Maldonado R, Puig MV and Robledo P (2019) Methylphenidate attenuates the cognitive and mood alterations observed in Mbnl2 knockout mice and reduces microglia overexpression. Cerebral Cortex 29(7), 2978–2997. 30060068 10.1093/cercor/bhy164 PMC7963113

[R53] Rommelse N, Buitelaar JK and Hartman CA (2017) Structural brain imaging correlates of ASD and ADHD across the lifespan: a hypothesis-generating review on developmental ASD-ADHD subtypes. Journal of Neural Transmission 124(2), 259–271. 28000020 10.1007/s00702-016-1651-1 PMC5285408

[R54] Rubia K (2011) Cool” inferior frontostriatal dysfunction in attention-deficit/hyperactivity disorder versus “hot” ventromedial orbitofrontal-limbic dysfunction in conduct disorder: a review. Biological Psychiatry 69(12), e69–e87. 21094938 10.1016/j.biopsych.2010.09.023

[R55] Rubia K (2018) ADHD brain function. In Banaschewski T, Coghill D and Zuddas A (ed), Oxford extbook of Attention Deficit Hyperactivity Disorder, Oxford University Press, pp. 62–93.

[R56] Samea F, Soluki S, Nejati V, Zarei M, Cortese S, Eickhoff SB, Tahmasian M and Eickhoff CR (2019) Brain alterations in children/adolescents with ADHD revisited: a neuroimaging meta-analysis of 96 structural and functional studies. Neuroscience & Biobehavioral Reviews 100, 1–8. 30790635 10.1016/j.neubiorev.2019.02.011 PMC7966818

[R57] Schalbetter SM, von Arx AS, Cruz-Ochoa N, Dawson K, Ivanov A, Mueller FS, Lin H-Y, Amport Ré, Mildenberger W, Mattei D, Beule D, Földy C, Greter M, Notter T and Meyer U (2022) Adolescence is a sensitive period for prefrontal microglia to act on cognitive development. Science Advances 8(9), eabi6672. 10.1126/sciadv.abi6672 PMC8890703 35235358

[R58] Sciberras E, Mulraney M, Silva D and Coghill D (2017) Prenatal risk factors and the etiology of ADHD—review of existing evidence. Current Psychiatry Reports 19(1), 1–8. 28091799 10.1007/s11920-017-0753-2

[R59] Shabab T, Khanabdali R, Moghaddamtousi SZ, Kadir HA and Mohan G (2017) Neuroinflammation pathways: a general review. International Journal of Neuroscience 127(7), 624–633. 27412492 10.1080/00207454.2016.1212854

[R60] Shaw P, Gilliam M, Liverpool M, Weddle C, Malek M, Sharp W, Greenstein D, Evans A, Rapoport J and Giedd J (2011) Cortical development in typically developing children with symptoms of hyperactivity and impulsivity: support for a dimensional view of attention deficit hyperactivity disorder. American Journal of Psychiatry 168(2), 143–151. 21159727 10.1176/appi.ajp.2010.10030385 PMC3268520

[R61] Shaw P, Szekely E, Banaschewski T, Coghill D and Zuddas A (2018) Insights from neuroanatomical imaging into ADHD throughout the lifespan. In Banaschewski T, Coghill D and Zuddas A (eds), Oxford extbook of Attention Deficit Hyperactivity Disorder, Oxford University Press, pp. 73–81.

[R62] Tayebati K, Tomassoni S, D F (2016) Neuroinflammatory markers in spontaneously hypertensive rat brain: An immunohistochemical study. CNS & Neurological Disorders-Drug Targets 15(8), 995–1000. 27238154 10.2174/1871527315666160527155014

[R63] Teissier A and Pierani A (2021) Wiring of higher-order cortical areas: spatiotemporal development of cortical hierarchy. Seminars in Cell & Developmental Biology 118, 35–49. 34034988 10.1016/j.semcdb.2021.05.010

[R64] Ugarte G, Piña R, Contreras D, Godoy F, Rubio D, Rozas C, Zeise M, Vidal R, Escobar J and Morales B (2023) Attention deficit-hyperactivity disorder (ADHD): from abnormal behavior to impairment in synaptic plasticity. Biology 12(9), 1241. 37759640 10.3390/biology12091241 PMC10525904

[R65] Uzay B, Bahadır-Varol A, Hökelekli FÖ, Yılmaz M, Esen EC, Başar K, Ayhan Y, Dalkara T and Eren-Koçak E (2024) FGF2 gene’s antisense protein, NUDT6, plays a depressogenic role by promoting inflammation and suppressing neurogenesis without altering FGF2 signalling. The Journal of Physiology 602(7), 1427–1442. 38468384 10.1113/JP285479

[R66] von Arx AS, Dawson K, Lin H-Y, Mattei D, Notter T, Meyer U and Schalbetter SM (2023) Prefrontal microglia deficiency during adolescence disrupts adult cognitive functions and synaptic structures: a follow-up study in female mice. Brain, Behavior, and Immunity 111, 230–246. 37100210 10.1016/j.bbi.2023.04.007

[R67] Wang Y, Hu Z, Liu H, Gu Y, Ye M, Lu Q, Lu X and Huang C (2022) Adolescent microglia stimulation produces long-lasting protection against chronic stress-induced behavioral abnormalities in adult male mice. Brain, Behavior, and Immunity 105, 44–66. 35781008 10.1016/j.bbi.2022.06.015

[R68] Yamashita M, Sakakibara Y, Hall FS, Numachi Y, Yoshida S, Kobayashi H, Uchiumi O, Uhl GR, Kasahara Y and Sora I (2013) Impaired cliff avoidance reaction in dopamine transporter knockout mice. Psychopharmacology 227(4), 741–749. 23397052 10.1007/s00213-013-3009-9

[R69] Yokokura M, Takebasashi K, Takao A, Nakaizumi K, Yoshikawa E, Futatsubashi M, Suzuki K, Nakamura K, Yamasue H and Ouchi Y (2021) In vivo imaging of dopamine D1 receptor and activated microglia in attention-deficit/hyperactivity disorder: a positron emission tomography study. Molecular Psychiatry 26(9), 4958–4967. 32439845 10.1038/s41380-020-0784-7

[R70] York EM, LeDue JM, Bernier L-P and MacVicar BA (2018) 3DMorph automatic analysis of microglial morphology in three dimensions from Ex Vivo and In Vivo imaging. eNeuro 5(6), ENEURO.0266–18.2018. 10.1523/ENEURO.0266-18.2018 PMC6325541 30627639

[R71] Yu M, Gao X, Niu X, Zhang M, Yang Z, Han S, Cheng J and Zhang Y (2023) Meta-analysis of structural and functional alterations of brain in patients with attention-deficit/hyperactivity disorder. Frontiers in Psychiatry 13, 1070142. 10.3389/fpsyt.2022.1070142 PMC9853532 36683981

[R72] Zhang L, Spencer TJ, Biederman J and Bhide PG (2018) Attention and working memory deficits in a perinatal nicotine exposure mouse model. PloS One 13(5), e0198064. 10.1371/journal.pone.0198064 PMC5967717 29795664

[R73] Zhang X, Sun D, Zhou X, Zhang C, Yin Q, Chen L, Tang Y, Liu Y and Morozova-Roche LA (2023) Proinflammatory S100A9 stimulates TLR4/NF-κB signaling pathways causing enhanced phagocytic capacity of microglial cells. Immunology Letters 255, 54–61. 36870421 10.1016/j.imlet.2023.02.008

[R74] Zhu J, Fan F, McCarthy DM, Zhang L, Cannon EN, Spencer TJ, Biederman J and Bhide PG (2017) A prenatal nicotine exposure mouse model of methylphenidate responsive ADHD-associated cognitive phenotypes. International Journal of Developmental Neuroscience 58(1), 26–34. 28179105 10.1016/j.ijdevneu.2017.01.014

[R75] Zhu J, Zhang X, Xu Y, Spencer TJ, Biederman J and Bhide PG (2012) Prenatal nicotine exposure mouse model showing hyperactivity, reduced cingulate cortex volume, reduced dopamine turnover, and responsiveness to oral methylphenidate treatment. Journal of Neuroscience 32(27), 9410–9418. 22764249 10.1523/JNEUROSCI.1041-12.2012 PMC3417040

